# Mohs surgical postoperative wound infection caused by rare Enterobacteriaceae, *Raoultella planticola*

**DOI:** 10.1016/j.jdcr.2025.06.031

**Published:** 2025-07-05

**Authors:** Jena C. Jacobs, Mario J. Sequeira

**Affiliations:** a A.T. Still University, Kirksville, Missouri; b Brevard Skin and Cancer Center, Rockledge, Florida; c University of Miami Miller School of Medicine, Miami, Florida

**Keywords:** dermatology, Enterobacteriaceae, infectious disease, microbiology, Mohs surgery, oncology, Raoultella planticola, surgical complications, wound care

## Introduction

*Raoultella planticola* (*R. planticola*) is a gram-negative, anaerobic, rod-shaped bacterium, belonging to the Enterobacteriaceae family.^1^ It was first found in humans in 1984 in a patient with sepsis and was originally classified as the *Klebsiella* spp., until 2001 when it was reclassified as a member of the *Raoultella* genus based on phylogenetic gene analysis.^2^ Initially it was considered a harmless environmental organism primarily found in soil and water.^2^ However, in recent years, it has emerged as a virulent pathogen closely associated with multiple severe human infections, including liver abscesses, cholangitis, pancreatitis, conjunctivitis, acute cholecystitis, urinary tract infections, and pneumonia.^3^ The reported cases involving skin infections were linked to a dog scratch, fatal bacteremia following burn wounds exposed to contaminated rainwater used to extinguish a flesh fire, a fractured tibia after an open reduction internal fixation, pelvic cellulitis in a neutropenic patient, and a crush injury of the thumb in a dirty environment washed with running water.^4^, ^5^, ^6^, ^7^, ^8^

In this study, we report an 85-year-old woman with a *R. planticola* wound infection of the right lower extremity following Mohs micrographic surgery. We aim to shed light on the rare potential for this organism to infect surgical wounds and cause delayed wound healing. To our knowledge, no cases of *R. planticola* have been reported in the setting of postsurgical wound infections following Mohs surgery.

## Case report

An 85-year-old woman presented to our Cancer Center on February 10, 2023 for Mohs micrographic surgical excision of a squamous cell carcinoma keratoacanthoma type, located on her right shin. Her past medical history included malignant melanoma, squamous cell carcinoma, basal cell carcinoma, seborrheic dermatitis, Grover’s disease, deep vein thrombosis, hyperlipidemia, and thyroid dysfunction. Her medications included ketoconazole 2% shampoo, levothyroxine 50 mcg, rosuvastatin calcium 40 mg, enoxaparin sodium 60 mg/0.6 mL, rivaroxaban 20 mg, and multivitamin. The patient drank 5 alcoholic beverages a day and had never smoked. Preoperative review of systems and physical examination were unremarkable; she appeared in good health, well nourished, and alert.

**Fig 1:**
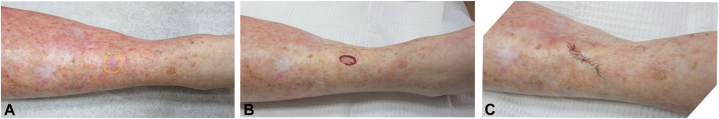
**A,** SCCKA, measuring 1.3 cm on the anterior right shin, denoted by *yellow circle*. **B,** Mohs defect, 1.7 cm. **C,** Final closed repair. *SCCKA*, Squamous cell carcinoma keratoacanthoma.

The surgical team conducted proper wound preparation using chlorhexidine scrub, sterile draping, and gloving. The squamous cell carcinoma keratoacanthoma, measuring 1.3 cm, was excised in a single stage, resulting in a 1.7-cm defect (Fig 1, *A* and *B*). Closure was achieved with 3 deep 4.0 polyglactin 910 sutures, one superficial interrupted and one running 4.0 nylon suture, resulting in a 2.7 cm linear repair (Fig 1, *C*). A Telfa nonadherent pad, mupirocin ointment, sterile gauze, and paper tape were applied as a pressure dressing.

**Fig 2:**
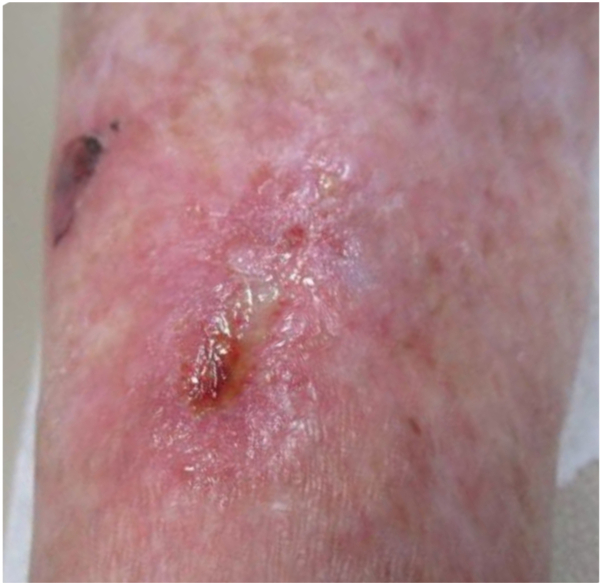
Infected surgical wound.

Fourteen days postsurgery, on February 24, 2023, the patient returned for suture removal. Examination revealed a dry, well-approximated wound with no erythema or drainage. However, 3 days later, on February 27, 2023, she reported abnormal wound healing. Physical examination revealed a superficial wound with mild erythema, granulation tissue, fibrinous slough, and an overlying crust. A scant amount of odorless, clear transudate was present, which was cultured, yielding heavy growth of *Raoultella planticola* (Fig 2) (Table I).

**Table I:** Bacterial culture and sensitivities

Culture, aerobic bacteria	
Micro number	16615949
Specimen source	Right shin
Specimen quality	Adequate
Result	Heavy growth of *Raoultella* (*Klebsiella*) *planticola*
Comment	Skin flora also present
Antibiotics	Susceptibility (MIC)
Amoxicillin/clavulanate	S/≤2 mcg/mL
Ampicillin	R/8 mcg/mL
Ampicillin/sulbactam	S/≤2 mcg/mL
Cefazolin	NR/≤4 mcg/mL ∗∗1
Cefepime	S/≤1 mcg/mL
Ceftazidime	S/≤1 mcg/mL
Ceftriaxone	S/≤1 mcg/mL
Ciprofloxacin	S/≤0.12 mcg/mL
Gentamicin	S/≤1 mcg/mL
Imipenem	S/0.5 mcg/mL
Levofloxacin	S/≤0.12 mcg/mL
Piperacillin/tazobactam	S/≤4 mcg/mL
Tobramycin	S/≤1 mcg/mL
Trimethoprim/sulbactam	S/≤20 mcg/mL

Therapy comments: Note ∗∗1: For infections other than uncomplicated UTI caused by *Escherichia coli*, *Klebsiella pneumonia*, or *Proteus mirabilis*, cefazolin is resistant of MIC > or = 8 mcg/mL (distinguishing susceptible vs intermediate for isolates with MIC ≤4 mcg/mL requires additional testing).

*I*, Intermediate; *MIC*, minimal inhibitory concentration; *NR*, not reported; *R*, resistant; *S*, susceptible.

**Fig 3:**
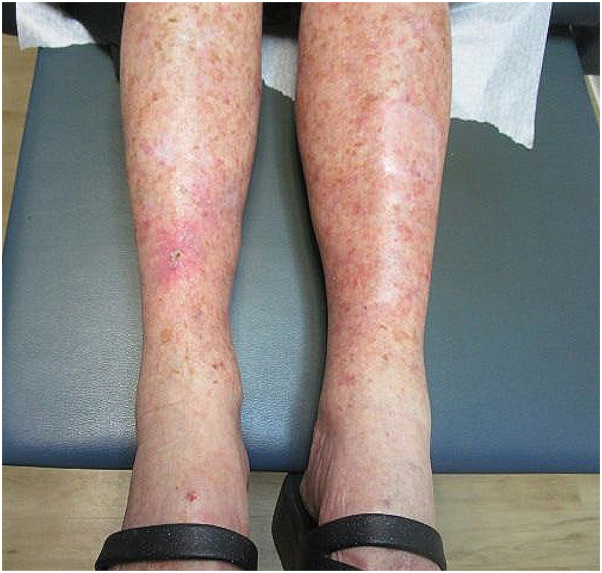
Healing wound with residual crust and postinflammatory redness.

Antibiotic susceptibility testing identified multiple effective treatments. The patient was prescribed oral amoxicillin/clavulanate (500/125 mg twice daily), topical gentamicin ointment (0.1% twice daily), along with aluminum acetate solution (Domeboro) daily soaks. By her follow-up on March 27, 2023, the oral antibiotic course was completed, but the wound displayed persistent granulation tissue and transudate. Wound debridement was performed, and daily cadexomer iodine gel (Iodosorb) application was initiated. The patient returned on April 03, 2023 with concerns of nonhealing wound, and a repeat culture identified only *Proteus mirabilis* sensitive to amoxicillin/clavulanate. A new course of amoxicillin-clavulanate (500/125 mg twice daily for 10 days), aluminum acetate solution soaks, and daily Iodosorb gel application were prescribed. On April 24, 2023, 10 weeks after the initial Mohs procedure, the wound was fully healed with no signs of infection (Fig 3).

## Discussion

This case adds to the limited literature on *R. planticola* as a rare but significant pathogen in wound infections, particularly following surgical interventions. This gram-negative organism is traditionally associated with environmental sources, such as contaminated water and soil, and is very infrequently implicated in human infections. Notably, *R. planticola* infections have been documented in cholangitis, urinary infections, and systemic bacteremia. However, cases involving skin infections remain extremely rare, especially in surgical wounds, as seen here. This patient’s history of malignancy and her lesion’s location on the lower extremity could have contributed to the infection, as certain regions, like the lower limbs, are more susceptible to delayed healing due to compromised vascularity and proximity to potential environmental contamination. Her hobby of gardening likely placed her at an increased risk. *R. planticola* strains are usually susceptible to third-generation or fourth-generation cephalosporins, Beta-lactamase inhibitor combinations, aminoglycosides, ciprofloxacin, levofloxacin, tigecycline, and carbapenems.^9^ Although *R. planticola* infections are rare, especially in dermatologic surgery, awareness of this pathogen’s potential role in wound infections is crucial. Early culture and sensitivity testing, along with tailored antibiotic therapy, can significantly improve patient outcomes by mitigating complications and reducing morbidity associated with delayed wound healing. This case also emphasizes the value of follow-up and reassessment in wound management, particularly when initial treatment fails to resolve the infection fully.

## Conflicts of interest

None disclosed.
